# Potential Use of MALDI-ToF Mass Spectrometry for Rapid Detection of Antifungal Resistance in the Human Pathogen *Candida glabrata*

**DOI:** 10.1038/s41598-017-09329-4

**Published:** 2017-08-22

**Authors:** Antonietta Vella, Elena De Carolis, Enrica Mello, David S. Perlin, Dominique Sanglard, Maurizio Sanguinetti, Brunella Posteraro

**Affiliations:** 1 Institute of Microbiology, Università Cattolica del Sacro Cuore, Fondazione Policlinico Universitario Agostino Gemelli, Largo F. Vito 1, 00168 Rome, Italy; 2 Public Health Research Institute, New Jersey Medical School, Rutgers Biomedical and Health Sciences, 225 Warren Street, Rutgers, Newark, New Jersey 07103 USA; 3 Institute of Microbiology, University Hospital of Lausanne, Rue du Bugnon 48, CH-1011 Lausanne, Switzerland; 4 Institute of Public Health (Section of Hygiene), Università Cattolica del Sacro Cuore, Fondazione Policlinico Universitario Agostino Gemelli, Largo F. Vito 1, 00168 Rome, Italy

## Abstract

The echinocandins are relatively new antifungal drugs that represent, together with the older azoles, the recommended and/or preferred agents to treat candidaemia and other forms of invasive candidiasis in human patients. If “time is of the essence” to reduce the mortality for these infections, the administration of appropriate antifungal therapy could be accelerated by the timely reporting of laboratory test results. In this study, we attempted to validate a MALDI-ToF mass spectrometry-based assay for the antifungal susceptibility testing (AFST) of the potentially multidrug-resistant pathogen *Candida glabrata* against anidulafungin and fluconazole. The practical applicability of the assay, reported here as MS-AFST, was assessed with a panel of clinical isolates that were selected to represent phenotypically and genotypically/molecularly susceptible or resistant strains. The data show the potential of our assay for rapid detection of antifungal resistance, although the MS-AFST assay performed at 3 h of the *in vitro* antifungal exposure failed to detect *C. glabrata* isolates with echinocandin resistance-associated *FKS2* mutations. However, cell growth kinetics in the presence of anidulafungin revealed important cues about the *in vitro* fitness of *C. glabrata* isolates, which may lead to genotypic or phenotypic antifungal testing in clinical practice.

## Introduction

Infections caused by *Candida* species are particularly severe in immunocompromised patients, including HIV-infected, cancer and transplant patients^1^. The severity of these infections, especially candidaemia and deep-seated tissue candidiasis—both are forms of invasive candidiasis^2^, is associated with a concerted interplay between antifungal drug resistance, virulence and immune evasion traits^3^. Among non-*albicans Candida* species, *C. glabrata* is the second (after *C. albicans*) or third most common cause of fungal bloodstream infections in the US^4, 5^. Outside of the US or Northern Europe^6^, *C. glabrata* is less commonly isolated^7^. Nonetheless, *C. glabrata* manifests an intrinsically low susceptibility to azoles *in vitro* or tends to easily develop azole resistance during the course of antifungal therapy^8, 9^. Alarmingly, exposure of *C. glabrata* cells to an antifungal agent may lead to highly variable, “evolvable” genomes capable of quick development of resistance to multiple antifungal drug classes^10^, including triazoles (*e.g*. fluconazole) and echinocandins (*e.g*. anidulafungin).

Despite a very low echinocandin resistance in many centres/countries, a recent US study has shown that nearly one-third of the *C. glabrata* isolates resistant to at least one echinocandin were also resistant to fluconazole^11^. Interestingly, almost all of these isolates with either a resistant or intermediate echinocandin minimum inhibitory concentration (MIC) value had mutations in specific hot spot regions (*e.g*. HS1) of the *FKS1* and *FKS2* genes^11^, which is the major mechanism of echinocandin resistance in *Candida* species^12, 13^. Such a mechanism is restricted and quite different from that of azoles^14^. Therefore, while direct molecular evaluation of the *FKS* genotype can be ideal for echinocandin resistance^15^, this is not the same for azole resistance due to the complexity of underlying resistance mechanisms^16^.

The possibility of using matrix-assisted laser desorption ionization–time-of-flight mass spectrometry (MALDI-ToF MS) for determination of antimicrobial resistance has been recently investigated^17^ to expand the landscape of diagnostic applications of this powerful technology^18^. In the present study, a phenotypically oriented assay using MALDI-ToF MS was developed and validated to allow accurate detection of susceptibility/resistance to anidulafungin and fluconazole in *C. glabrata* isolates after antifungal exposure *in vitro*. The results suggest that the MALDI-ToF MS-based AFST assay, reported here as MS-AFST, has the potential to become a useful tool for rapid detection of antifungal resistance.

## Results

Recently, we have proposed a simplified version of the MS-AFST assay^19^ that allowed successful discrimination between susceptible and resistant isolates of *C. albicans* only after 3 h of incubation of yeast cells in the presence of three concentrations of echinocandin (*i.e*. caspofungin): no drug (null concentration), intermediate (“breakpoint” concentration) and maximum (maximal concentration)^20^.

In this study, *C. glabrata* was chosen as a model test organism for the validation of MS-AFST against anidulafungin and fluconazole and the practical applicability of MS-AFST was demonstrated with a panel of 80 *C. glabrata* clinical isolates selected to represent phenotypically and genotypically/molecularly susceptible or resistant strains (Supplementary Table 1).

### Optimizing MALDI-ToF MS analysis

First, we set up experiments with 10 *C. glabrata* isolates representative of wild-type (WT; without acquired mutational or other resistance mechanisms) or non-wild-type (NWT; with acquired mutational or other resistance mechanisms) phenotypes (Supplementary Table 1). Following exposure for 3 h to anidulafungin or fluconazole, mass spectra obtained from these isolates were subjected to composite correlation index (CCI) analysis—a CCI value near 1.0 indicates relatedness between the spectral sets, and 0 indicates no match—in order to identify, respectively, the “breakpoint” concentration spectrum to be compared with the spectra at two extreme drug concentrations (null or maximal). As a result, 0.06 µg/mL for anidulafungin and 16 µg/mL for fluconazole were identified as the respective concentrations at which the CCI values obtained by matching the “breakpoint” spectrum with the spectrum at maximal drug concentration (maximum CCIs) were, respectively, higher (for all the 5 WT isolates) or lower (for all the 5 NWT isolates) than the CCI values obtained when the “breakpoint” spectrum was matched with the spectrum at null drug concentration (null CCIs).

The reproducibility of MS-AFST assays was assessed for the 10 reference isolates tested on two different days. In all the analyses, CCI values from the isolates’ biological replicates differ uniformly (*p* < 0.05, unpaired t-test). Although absolute CCI values (maximum and null) varied among the two replicates, the CCI ratios expressed as CCI_max_/CCI_null_ were always higher than 1 for the WT isolates and lower than 1 for the NWT isolates (data not shown). The classification results by the MS-AFST assays were consistent with the isolates’ MIC values and the respective susceptibility categories—as established by the Clinical and Laboratory Standards Institute (CLSI)—shown in Supplementary Table 1.

### Using MS-AFST assay for discrimination between anidulafungin-resistant and anidulafungin-susceptible isolates

Table 1 shows the classification results for all the 80 *C. glabrata* isolates included in the study, which were tested with the MS-AFST assay using the three established concentrations (0, 0.06 and 32 µg/mL) of anidulafungin.

**Table 1:** MS-AFST assay results for the 80 *C. glabrata* isolates tested against anidulafungin.

Genotype (no. of isolates)	MIC range, expressed as μg/mL	Classification by MS-AFST assay (no. of isolates)
WT (58)	≤0.016–1	Susceptible (57), resistant (1)
***FKS1***		
F625S (2)	1–2	Resistant (2)
S629P (2)	2–4	Resistant (2)
D632E (2)	1	Resistant (2)
***FKS2***		
F659V (2)	1	Susceptible (2)
S663P (8)	0.25–4	Resistant (3), susceptible (5)
R665G (1)	0.25	Susceptible (1)
R665S (1)	0.12	Susceptible (1)
D666E (1)	0.25	Susceptible (1)
D666G (1)	2	Resistant (1)
P667T (2)	0.25–1	Susceptible (2)

Classification of the isolates as susceptible or resistant to the antifungal drug is shown according to the *FKS* HS1 genotypes and MIC values.

**Figure 1:**
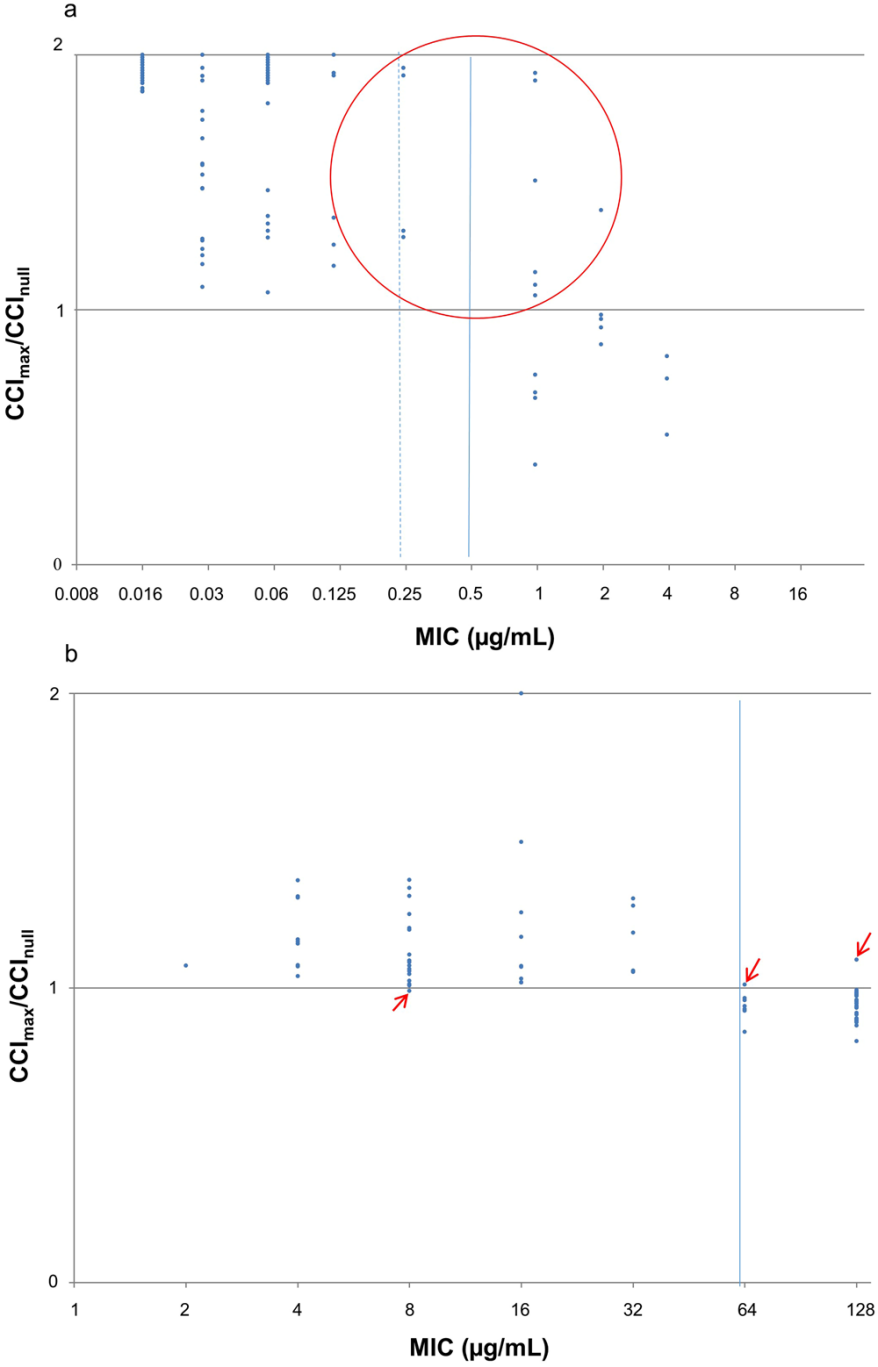
Distribution of results from the MS-AFST assay for the 80 *C. glabrata* isolates according to the MIC values as determined by the reference CLSI method. The isolates were tested against the antifungal drugs anidulafungin (**a**) and fluconazole (**b**). For each isolate, the MIC value (*x*-axis) was plotted against the CCI_max_/CCI_null_ ratio (*y*-axis). The latter was calculated as the maximum CCI value (*i.e*. the value obtained by matching the breakpoint spectrum with the spectrum at maximal drug concentration) divided by the null CCI value (*i.e*. the value obtained by matching the breakpoint spectrum with the spectrum at null drug concentration). Isolates were called susceptible if the CCI_max_/CCI_null_ ratio was higher than 1, whereas isolates were called resistant if the CCI_max_/CCI_null_ ratio was lower than 1. In the (**a**) and (**b**) graphs, solid lines indicate the MIC breakpoints for resistance to anidulafungin (≥0.5 µg/mL) and fluconazole (≥64 µg/mL), whereas the dotted line indicates the MIC breakpoint for the intermediate (0.25 µg/mL) susceptibility to anidulafungin. In (**a**) the red circle denotes isolates with MICs in the intermediate (n = 4) or resistance (n = 7) range to anidulafungin, whereas in (**b**) red arrows indicate single isolates with MICs in the dose-dependently susceptibility (n = 1) or resistance (n = 2) range to fluconazole; all the 11 isolates (8 for anidulafungin and 3 for both anidulafungin and fluconazole) had classification results by the MS-AFST assay that did not agree with the categorization results obtained by the reference CLSI method.

Overall, 86.2% (69/80) of the isolates were classified as susceptible and 13.8% (11/80) of the isolates were classified as resistant to anidulafungin. According to *FKS1*/*FKS2* HS1 genotypes, 57 (98.3%) of 58 isolates with WT sequences were classified as susceptible, 6 (100%) of 6 isolates with mutated *FKS1* sequences were classified as resistant, and 4 (25.0%) of 16 isolates with mutated *FKS2* sequences were classified as resistant. According to CLSI MIC breakpoints, 58 (100%) of 58 isolates in the susceptible category were classified as susceptible, and 11 (50.0%) of 22 isolates in the nonsusceptible (intermediate or resistant) category were classified as resistant. As shown in Fig. 1a, among 11 isolates with a CCI_max_/CCI_null_ ratio <1 (*i.e*. MS-AFST assay-classified as resistant), 4 isolates had MICs of 1 µg/mL, 4 isolates had MICs of 2 µg/mL, and 3 isolates had MICs of 4 µg/mL; among 69 isolates with a CCI_max_/CCI_null_ ratio >1 (*i.e*. MS-AFST assay-classified as susceptible), 58 isolates had MICs ranging from 0.016 to 0.125 µg/mL, 4 isolates had MICs of 0.25 µg/mL, and 7 isolates had MICs ranging from 1 to 2 µg/mL.

### Using MS-AFST assay for discrimination between fluconazole-resistant and fluconazole-susceptible isolates

Table 2 shows the classification results for the aforementioned 80 *C. glabrata* isolates that were tested with the MS-AFST assay using the three established concentrations (0, 16 and 256 µg/mL) of fluconazole.

**Table 2:** MS-AFST assay results for the 80 *C. glabrata* isolates tested against fluconazole.

*CDR1*/*CDR2* expression (no. of isolates)	MIC range, expressed as μg/mL	Classification by MS-AFST assay (no. of isolates)
Basal (41)	2–32	Susceptible (40), resistant (1)
Increase (39)	64–>64	Resistant (37), susceptible (2)

Classification of the isolates as susceptible or resistant to the antifungal drug is shown according to the *CDR* expression levels and MIC values.

Overall, 52.5% (42/80) of the isolates were classified as susceptible and 47.5% (38/80) of the isolates were classified as resistant to fluconazole. According to the underlying resistance mechanism, 40 (97.6%) of 41 isolates with a basal level of *CDR1*/*CDR2* expression were classified as susceptible, whereas 37 (94.9%) of 39 isolates with an increased level of *CDR1*/*CDR2* expression were classified as resistant. According to CLSI MIC breakpoints, 40 (97.6%) of 41 isolates in the susceptible dose-dependent category were classified as susceptible, and 37 (94.9%) of 39 isolates in the resistant category were classified as resistant. As shown in Fig. 1b, among 38 isolates with a CCI_max_/CCI_null_ ratio <1 (MS-AFST assay-classified as resistant), 37 isolates had MICs ranging from 64 to 128 µg/mL, and 1 isolate had a MIC of 8 µg/mL; among 42 isolates with a CCI_max_/CCI_null_ ratio >1 (MS-AFST assay-classified as susceptible), 40 isolates had MICs ranging from 2 to 32 µg/mL, and 2 isolates had MICs ranging from 64 to 128 µg/mL.

### Resolving the misclassification errors obtained with MS-AFST assay

The performances of both MS-AFST assay and CLSI method for the 80 *C. glabrata* isolates were compared to the antifungal-resistance molecular analysis results (Supplementary Table 2). As many as 11 very major errors (*i.e*. if a resistant isolate was misclassified as susceptible) occurred when testing anidulafungin, whereas only 2 very major errors did when testing fluconazole, with the MS-AFST assay. Interestingly, 3 of the 11 errors concerned the isolates DSP220, DSP221 and DSP259, which were misclassified as resistant (1 isolate) or susceptible (2 isolates) against fluconazole. The major error (*i.e*. if a susceptible isolate was misclassified as resistant) with anidulafungin for the isolate DSP182 was not further evaluated because it was also considered resistant by the CLSI method.

Therefore, we performed MS-AFST-assays with the 11 isolates incubated in the presence of antifungal drugs for times higher than 3 h (*i.e*. 6-, 9- and 12-h incubations; see Supplementary Table 3). Two errors (only for anidulafungin) regarding isolates DSP282 and DSP314 were still unresolved. However, when MS-AFST assays were performed at 15 h of incubation with both the antifungal drugs, we found 100% essential agreement between minimal profile change concentration (MPCC) values and MICs for all the 11 isolates (Supplementary Table 3).

### Determining *in vitro* growth kinetics to reveal differences between *FKS1* and *FKS2* mutant isolates

To investigate whether the different capability of the MS-AFST assay in discriminating among *C. glabrata* isolates with mutated *FKS1* or *FKS2* genotypes might be reflected by a differentially defective cell growth, *in vitro* growth of 24*C. glabrata* isolates (2 *FKS1*/*FKS2*-WT; 6 *FKS1*-NWT and 16 *FKS2*-NWT) was recorded in the presence of anidulafungin.

**Figure 2:**
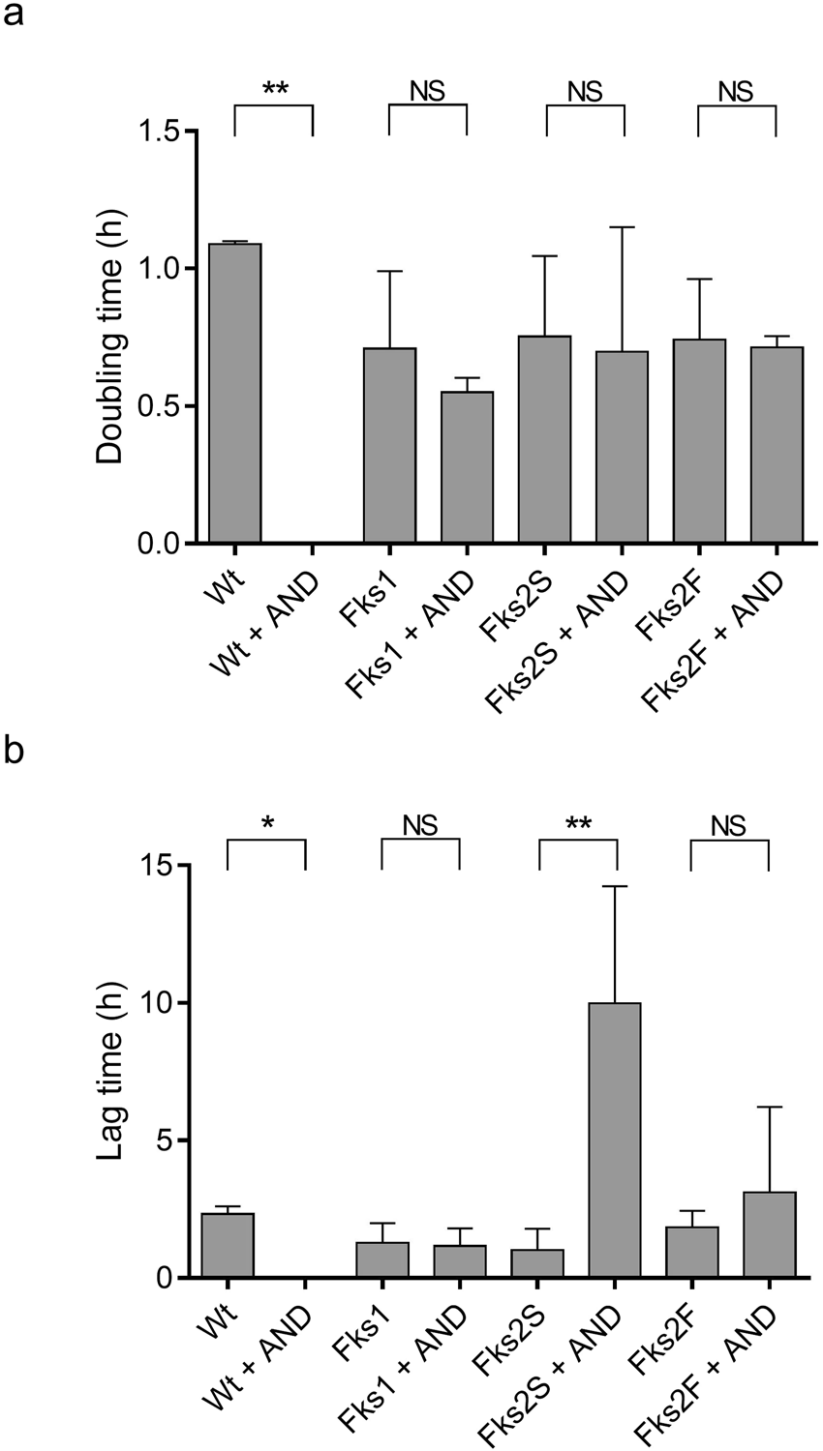
Cell growth parameters determined by an algorithm-based automatic calculation for *C. glabrata* isolates exposed to anidulafungin. A total of 24 isolates, 2 with WT *FKS* genes, 6 with NWT *FKS1* genes and 16 with NWT *FKS2* genes were compared. For each group, growth parameters were evaluated in the isolates exposed to 0.06-µg/mL anidulafungin (AND) in comparison to the same isolates that were not exposed to the antifungal drug. Two subgroups of *FKS2*-NWT_fast_ and *FKS2*-NWT_slow_ isolates, *i.e*. identified within the *FKS2*-NWT isolates based on marked differences in their cell growth profiles, were also compared. The cell doubling times (in h) are measured at the maximal growth rate. The lag times (in h) represent the mean ± SD of three independent growth curves of cells exposed at the same time.

The growth curves of *C. glabrata* isolates DSP2 (*FKS1*/*FKS2*-WT), DSP224 (*FKS1*-S629P), DSP34 (*FKS2*-P667T) and DSP186 (*FKS2*-S663P) are shown, as representative examples, in Supplementary Fig. 1 and Fig. 2. Overall, growth curves obtained from the isolates in the presence or absence of anidulafungin were superimposable for both WT and *FKS1*-NWT isolates but not for *FKS2*-NWT isolates. For these last isolates, two growth profiles could be noticed, of which one very similar to that the *FKS1*-NWT isolates and another quite different from that of WT or *FKS1*-NWT isolates—thus isolates corresponding to the two profiles were designated as *FKS2*-NWT_fast_ and *FKS2*-NWT_slow_, respectively.

Next, the cell doubling times of anidulafungin-exposed and unexposed isolates’ cells were compared among groups of *FKS1*-NWT or *FKS2*-NWT isolates (Fig. 2). Overall, no statistically significant differences in the mean doubling-time values were noticed (*p* > 0.05, for all comparisons; paired t-test) (Fig. 2a), whereas the mean lag-time values differed considerably (Fig. 2b). Interestingly, among the *FKS2*-NWT isolates, the *FKS2*-NWT_slow_ subgroup displayed mean lag-time values that were higher than those of the *FKS2*-NWT_fast_ subgroup (9.9 ± 4.2 h *vs* 3.1 ± 1.5 h, *p* < 0.05; unpaired t-test). These times were sufficiently prolonged to allow the *FKS2*-NWT_fast_ isolates to exhibit a profile of expressed proteins enabling them to be correctly classified by the MS-AFST assay.

## Discussion

In the present study, we were able to detect changes in the protein spectrum of *C. glabrata* in the presence or absence of anidulafungin or fluconazole that correlate with drug susceptibility changes. Overall, applying the 3 h MS-AFST assay on a panel of 80 *C. glabrata* isolates, we detected 85.0% (68/80) and 96.2% (77/80) of isolates with results of classification for anidulafungin and fluconazole, respectively, that were in full agreement with those obtained by antifungal-resistance mechanisms (*FKS1*/*FKS2* genotype or (*Cg*)*CDR1*/*CDR2* overexpression). Duplicate testing of each isolate on different runs against anidulafungin or fluconazole was performed to evaluate the robustness of the dual MS-AFST assay. Reproducibility rates of 98.7% and 97.5% were obtained when testing anidulafungin and fluconazole, respectively, with only 3 inconsistencies (1 for anidulafungin and 2 for fluconazole)—subsequently arbitrated by a third run before including them in the final analysis (see Materials and Methods). These data allow us to be confident about the accuracy of MS-AFST assays, although our results differ in some respects from those of previous investigators. In a recent evaluation of our assay by Saracli *et al*.^21^—the authors employed a modification of the assay originally developed by our laboratory^19^—the reproducibility of the MALDI-ToF MS-based assay for discrimination of susceptible and resistant isolates of *Candida* species to triazoles varied between 54.3% and 82.9%. However, consistent with our study, the reproducibility was higher for *C. glabrata* isolates (77.1% for fluconazole) than for isolates from other *Candida* species^21^.

When the MS-AFST assay results for anidulafungin were analysed according to the *FKS1* or *FKS2* genotype, we found 100% and 25.0% agreement for isolates (6/6) with a mutated *FKS1* gene and for isolates (4/16) with a mutated *FKS2* gene, respectively. Accordingly, 15.0% of incorrect classification results for anidulafungin were assigned to *FKS2* HS1 mutations, and these results were distributed among all 7 different mutated *FKS2* genotypes included in this study. In particular, we noticed that the isolates harbouring the mutations F659V (2 isolates), R665G (1 isolate), R665S (1 isolate), D666E (1 isolate) and P667T (2 isolates) were classified as anidulafungin-susceptible, whereas 1 isolate harbouring the D666G mutation and 3 of 8 isolates harbouring the S663P mutation were all classified as anidulafungin-resistant by the MS-AFST assay. However, the above overall rates of 85.0% and 96.2% increased to 97.5% and 100% for anidulafungin and fluconazole, respectively, when the 11 of 79 isolates—(excluding the isolate DSP182)—with incorrect classifications (8 to anidulafungin and 3 to both anidulafungin and fluconazole) were tested by prolonged antifungal drug exposures (up to 6–12 h). Two of 11 isolates, DSP282 (*FKS2*-R665G) and DSP314 (*FKS2*-P667T), were still not correctly classified by the MS-AFST assay, although their MPCC values—determined at a 15-h anidulafungin exposure—were identical to the CLSI MIC values (0.25 µg/mL). Together, this indicates that incubation times ranging between 12 and 15 h allow to correctly identify these isolates with our assay. Unfortunately, the 3 h AFST format assay in the presence of echinocandin failed to accurately detect *C. glabrata* isolates with *FKS2* mutations. Thus, the goal/benefit of providing reliable echinocandin resistance/susceptibility information on the same day was only partially reached with this specific antifungal agent in *C. glabrata*.

To attempt to elucidate the above deficiencies of the MS-AFST assay, 22 isolates with *FKS* mutations and 2 *FKS*-WT isolates were analysed for quantitative changes in cell growth under exposure to anidulafungin. We used a micro-culture growth curve assay in the presence of 0.06 µg/mL of anidulafungin (the “breakpoint” concentration used in the MS-AFST assay). This allowed us to profile growth with the lag times and subsequent growth rates. We found that the lag times in response to anidulafungin differed between *FKS1*-NWT isolates and *FKS2*-NWT isolates, and these differences were particularly marked between two subgroups of *FKS2*-NWT isolates. Indeed, among the *FKS2*-NWT_slow_ isolates, 1 isolate (DSP235; MIC, 0.125 µg/mL) had a growth profile similar to that of the WT isolates, and other 2 isolates (the aforementioned DSP282 and DSP314; MIC, 0.25 µg/mL) did not grow under the experimental conditions—likely due to problems intrinsic to the micro-culturing^22^. Excluding the 3 isolates, the 9 *FKS2*-NWT_slow_ isolates were greatly slowed in comparison with the 4 *FKS2*-NWT_fast_ isolates (DSP30, DSP32, DSP186 and DSP249), consistent with the observation that the latter isolates did reach in short times (*i.e*. ≤3 h) a cell protein expression level enough to be correctly classified by the MS-AFST assay. Notably, the mean lag time of *FKS2*-NWT_fast_ isolates was very similar to that of the *FKS1*-NWT isolates (1.28 *vs* 1.84, *p* = 0.476). Also, we were not surprised to find that all the 9 *FKS2*-NWT_slow_ isolates gave a correct classification result by the MS-AFST assay only after prolonged times (*i.e*. >3 h) of anidulafungin exposure. Since differences in cell growth between the two subgroups did not seem to be affected by the type of *FKS2* mutation, a strain-specific genetic background, overwhelming the effect of the mutated *FKS2* genotype, may explain the initially incorrect classification results by the MS-AFST assay.

**Figure 3:**
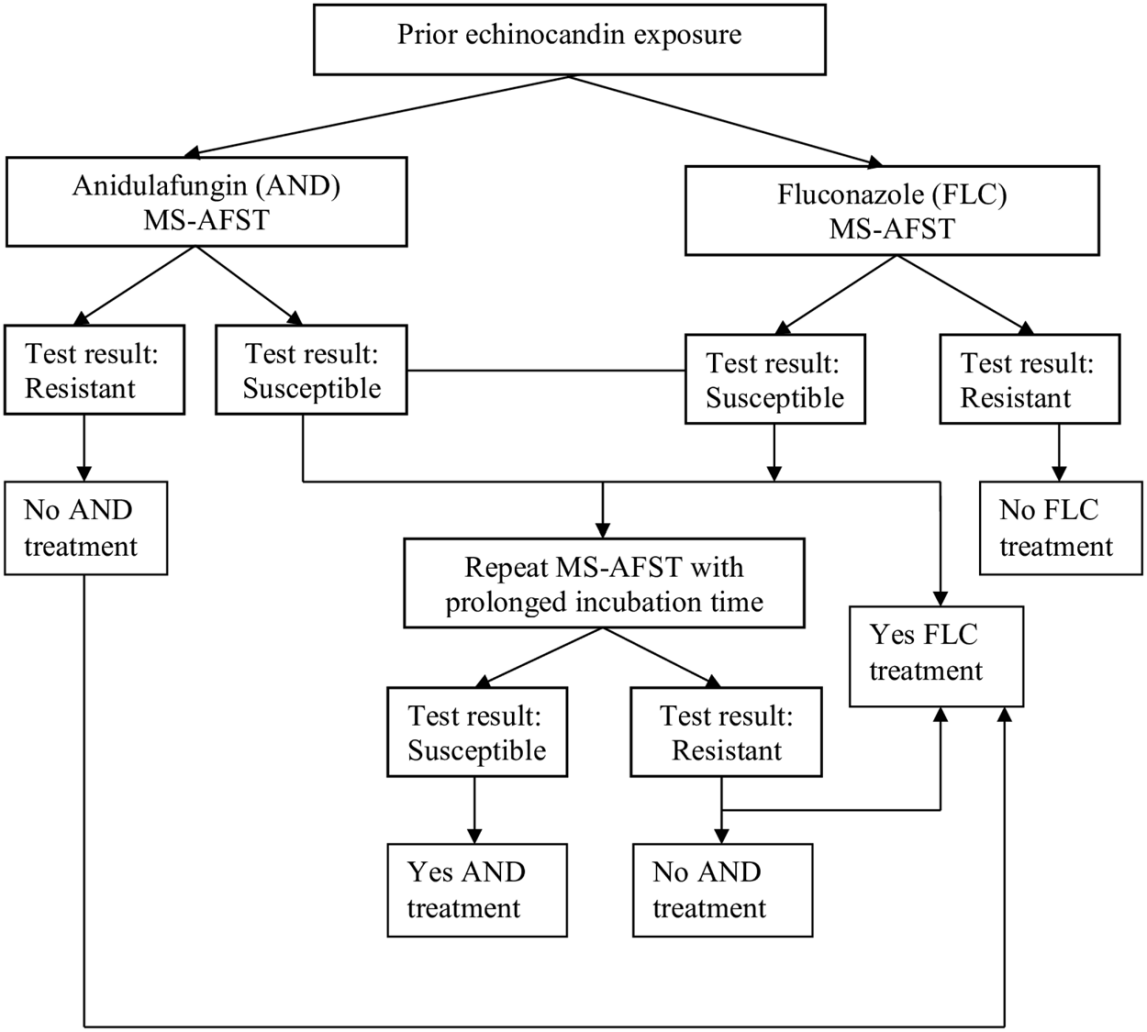
Flow chart diagram illustrating the potential use of the dual MS-AFST in clinical practice. A scenario of prior echinocandin exposure is shown, where MS-AFST results for a *C. glabrata* clinical isolate as susceptible or resistant to anidulafungin (AND) and/or fluconazole (FLC) may be useful to drive the appropriate administration of antifungal therapy. For AND, valid MS-AFST results can be available after 3 h or, in cases of isolates with *FKS2* HS1 mutations, 6–12 h of antifungal exposure *in vitro*.

In conclusion, our study is the first to evaluate the use of the MS-AFST assay on *C. glabrata* isolates with anidulafungin and fluconazole. The method in its quick version (3 h format) was highly specific for identifying isolates with altered *FKS1* gene sequences and/or *CDR1*/*CDR2* gene expression levels. Although no controls with other *Candida* species were included, our previous study with *C. albicans* and caspofungin suggests that *FKS1* can be considered an easily attainable target for the rapid assessment of echinocandin resistance. As above mentioned, we have shown that MS-AFST correctly classified 100% (51/51) and 90.9% (10/11) of WT and *FKS1* mutant isolates of *C. albicans* as caspofungin-susceptible and caspofungin-resistant, respectively^20^. Future studies are needed to validate our findings. In the meanwhile, as clinical experience suggests to use alternative antifungal classes for treating patients with previous echinocandin exposure, our MALDI-ToF MS assay, as shown in Fig. 3, could be used as a valuable tool for rapid detection of *C. glabrata* isolates as fluconazole-resistant, which could alert about the potential presence of anidulafungin resistance in these isolates.

## Materials and Methods

### Yeast organisms and growth conditions

A total of 80 *C. glabrata* isolates was included in the study, that comprised WT and NWT isolates—the latter with *FKS1*/*FKS2* mutations and/or *CDR1*/*CDR2* overexpression—mostly recovered from clinical specimens. The isolates were retrieved from strain collections in the Perlin (DSP isolates), Sanglard (DSY or SFY isolates) or Sanguinetti (UCSC isolates) laboratories at the Public Health Research Institute, the University Hospital of Lausanne and the Università Cattolica del Sacro Cuore, Fondazione Policlinico Universitario Agostino Gemelli, respectively. As detailed in Supplementary Table 1, the set of isolates consisted of anidulafungin-susceptible, anidulafungin-intermediate, anidulafungin-resistant, fluconazole-susceptible dose-dependent and fluconazole-resistant isolates, and 11 of these isolates were resistant (nonsusceptible) to both anidulafungin and fluconazole. All the isolates were reanalysed for AFST before to be utilized in this study (see below). The mutations in HS1 of *FKS1* and *FKS2* genes were detected by DNA sequencing as reported elsewhere^23^, whereas the overexpression of (*Cg*)*CDR1* and (*Cg*)*CDR2* genes was assessed by quantitative real-time PCR analysis using the primers and thermal conditions previously described^24, 25^. Prior to testing, isolates were grown on YPD (yeast extract/peptone/dextrose) agar plates, unless otherwise specified.

### AFST by the reference method

Susceptibility testing of *C. glabrata* isolates to anidulafungin and fluconazole was performed according to the CLSI M27-A3 guidelines^26^, using microdilution-broth trays custom prepared with pure antifungal substances (anidulafungin provided by Pfizer, New York, NY; fluconazole provided by Sigma-Aldrich, St. Louis, MO) in the UCSC laboratory. Results were read after 24-h incubation of trays to determine, for each isolate, the MIC to antifungal drug as the lowest concentration of drug that caused a 50% growth reduction compared to the drug-free control well. The MICs to both the antifungals were interpreted using the 2012 CLSI M27-S4 breakpoints^27^. For anidulafungin, isolates were categorized as susceptible (MIC, ≤0.125 µg/mL), intermediate (MIC, 0.25 µg/mL) or resistant (≥0.5 µg/mL); however, for comparison purposes, isolates with MICs in the intermediate or resistance range to anidulafungin were conceptually placed into a nonsusceptible category, and thus considered as resistant. For fluconazole, isolates with an MIC of ≤32 µg/mL were categorized as susceptible dose-dependent, whereas isolates with an MIC of ≥64 µg/mL were categorized resistant; however, for comparison purposes, isolates with MICs in the susceptible dose-dependent range was considered as susceptible. This was in keeping with acceptance criteria published elsewhere^21, 28^.

### MALDI-ToF MS assays, data analysis, and reproducibility assessment

Samples for MALDI-ToF MS were prepared in duplicate by following analytical methods previously developed by us^19, 20^. In the first study phase, yeast cells (1 × 10^7^ CFU/mL) were exposed, in RPMI, to six different antifungal-drug concentrations (0.03 µg/mL to 16 µg/mL for anidulafungin, and 4 µg/mL to 128 µg/mL for fluconazole), and two additional antifungal-drug concentrations such as a maximal concentration (32 µg/mL for anidulafungin and 256 µg/mL for fluconazole) and a null concentration (0 µg/mL). After establishing the breakpoint antifungal-drug concentration^20^, in the second study phase, yeast cells (1 × 10^7^ CFU/mL) were exposed to the following antifungal-drug concentrations: 32 µg/mL, 0.06 µg/mL and 0 µg/mL for anidulafungin, and 256 µg/mL, 16 µg/mL and 0 µg/mL for fluconazole. After 3 h at 37 °C under agitation, cells were harvested by centrifugation, washed twice with deionized water, and re-suspended in 10% formic acid. One microliter of the lysate from each sample was used for the analysis which was performed, in duplicate, with the Bruker Daltonics MALDI Biotyper 3.1 software on a Microflex LT mass spectrometer (Bruker Daltonics, Bremen, Germany). The protein mass spectra were acquired in the positive linear mode and analysed in the mass range of 4,000 to 9,000 *m*/*z*, starting from measuring a larger mass range (2,000 to 20,000 *m*/*z*), by using 240 laser shots (40 laser shots at 6 different spot positions) for each spectrum and by instrument parameters previously established (ion source 1, at 20 kV; ion source 2, 16.7 kV; and lens, 8.5 kV)^20^. Before each run, the instrument was calibrated with Bacterial Test Standard (Bruker Daltonics).

The MALDI Biotyper 3.1 software was used for comparing the spectra with one another at the antifungal drug concentrations tested, as above mentioned, by means of the automatic visualization of raw spectra in a CCI matrix view, where CCI values around 1 represent high similarity of spectra and CCI values near 0 indicate clear diversity of the spectra. Based on previous studies from our group^19, 20^, the maximum CCI (CCI_max_) value and the null CCI (CCI_null_) value were computed, respectively, by matching each breakpoint spectrum with the spectrum at the maximal concentration of or with the spectrum at null concentration of antifungal drug. In addition, CCI ratios were obtained by dividing the CCI_max_ by the CCI_null_ and expressed as CCI_max_/CCI_null_, and were then used as a measure to evaluate the spectral variation within the single isolate.

As a first step of evaluation, the reproducibility of the analytical method was assessed with the spectra obtained from two biological replicates (*i.e*. prepared from cultivations of the same isolate on two different days) for representative isolates, which were selected from the isolate set of the study as specified above. In a next step, samples from all the study isolates were blind-coded, extracted, and subjected to MALDI-ToF MS analysis in two different days, and the results of the analyses were compared. The MS-AFST MS assay was considered reproducible when the same classification assessment was obtained after repeat testing regardless of its agreement with the categorical result obtained with the method used as a comparator (see below). In cases of inconsistency between the replicates, the analysis was repeated once again—this occurred in 1 case for anidulafungin and 2 cases for fluconazole, accounting for reproducibility rates of 98.7% and 97.5%, respectively. In these cases, concordant results of two of three runs were accepted for the final analysis. In addition, MALDI-ToF MS assays were also performed according to the original version of our AFST method to determine the MPCC to anidulafungin for those isolates that had discordant results with those of the comparator method (see below).

### Analysis of test results

Results from the MS-AFST assay were interpreted according to pre-definite criteria, by which a *C. glabrata* isolate was classified as susceptible or resistant to the drug if the CCI value derived from the correlation of spectra at the breakpoint and the maximal drug concentrations was, respectively, higher or lower than the CCI value derived from the correlation of spectra at the breakpoint and null drug concentrations^20^. The *FKS1*/*FKS2* genotype (for anidulafungin) or the *CDR* overexpression (for fluconazole) was used as a gold standard to calculate the percentage categorical agreement as a means of evaluating the performance of the MS-AFST assay. Very major errors were recorded when NWT isolates were classified as susceptible by the MS-AFST assay, and major errors were recorded when WT isolates were classified as resistant by the MS-AFST assay. Minor errors were not recorded because an intermediate category was not available by the MS-AFST assay. The performance of the CLSI method was evaluated in parallel as described for the MS-AFST assay. In addition, categorical agreement as well as very major or major errors of the MS-AFST assay were evaluated by comparison with the results from the CLSI method.

### *In vitro* cell growth analysis

To quantitate *in vitro* growth changes in *C. glabrata* isolates following exposure to anidulafungin, a methodology based on the micro-cultivation of yeast cells in liquid media was used^22^. By this way, pre-prepared growing media into microplates are inoculated with very small (100 µL) aliquots of pre-cultures (starting from single colonies and incubated overnight), and cell population is automatically monitored at regular time intervals for 48 h. In this study, *C. glabrata* isolates were grown exponentially to a density of 0.7 × 10^7^ to 10^7^ cells/mL (optical density [OD] at 660 nm  ~0.5–0.7) in YPD medium at 30 °C under constant agitation. Then, cells were harvested by centrifugation, washed with sterile water and re-suspended to a density of 0.8 × 10^7^ cells/mL. For each isolate, triplicates of wells containing 95 µL of YPD medium with 0.06-µg/mL anidulafungin (treated samples) or without anidulafungin (control samples) were each inoculated with 5 µL (4 × 10^4^ cells) of the washed cell suspension in a 96-well microplate. Three wells for the blank, with no cells in them, were included in the microplate. The cell population was monitored with a microplate scanning spectrophotometer (BioTek Absorbance Microplate Reader; Winooski, VT) at 30 °C. The OD of cell population was recorded every 15 min during 48 h at the wavelength of 630 nm, by using the Gen5 Reader Control Software of the BioTek instrument. The data were exported to Excel (Microsoft) and the raw OD curves were plotted to derive growth curves. For data quantification, we followed a well-established algorithm^22^ that allows the automatic calculation of growth parameters. In this algorithm, cell growth is represented by the logarithmic curve, Ln (OD/ODi), where ODi is the initial OD; the maximal growth rate (μ_m_) corresponds to the maximal slope of the Ln curve, and the cell doubling time at μ_m_ is defined by ln(2)/μ_m_; ultimately, the lag time (λ) corresponds to the intersection of the maximal slope of the Ln curve with the *x*-axis.

### Guidelines and regulations

No human subjects or human tissue were used in this study, therefore no human consent was required.

### Statistical analysis

The data were expressed as the mean ± the standard deviation (SD). Statistical significance was assessed using paired or unpaired t-tests. A *p* value of less than 0.05 was considered significant.

## Electronic supplementary material

Supplementary Information
