# Supplementary material for: Potential Use of MALDI-ToF Mass Spectrometry for Rapid Detection of Antifungal Resistance in the Human Pathogen Candida glabrata
Source: Sci Rep. 2017 Aug 22;7:9099. doi: 10.1038/s41598-017-09329-4 (PMC5567316; doi:10.1038/s41598-017-09329-4)
Supplement: Supplementary file 1 — Supplementary Information [file 41598_2017_9329_MOESM1_ESM.doc]

**Potential Use of MALDI-ToF Mass Spectrometry for Rapid Detection of Antifungal Resistance in the Human Pathogen *Candida glabrata***

Antonietta Vella, Elena De Carolis, Enrica Mello, David S. Perlin, Dominique Sanglard, Maurizio Sanguinetti & Brunella Posteraro

**
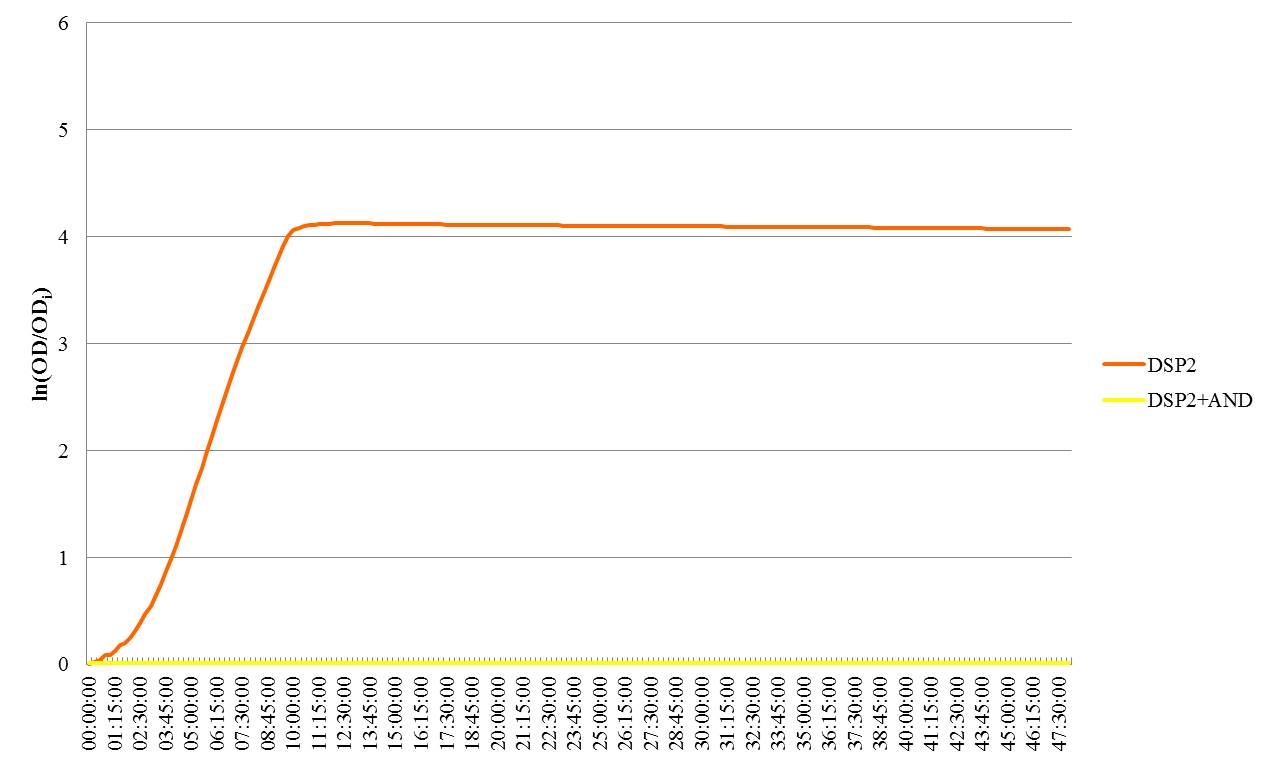
**

**
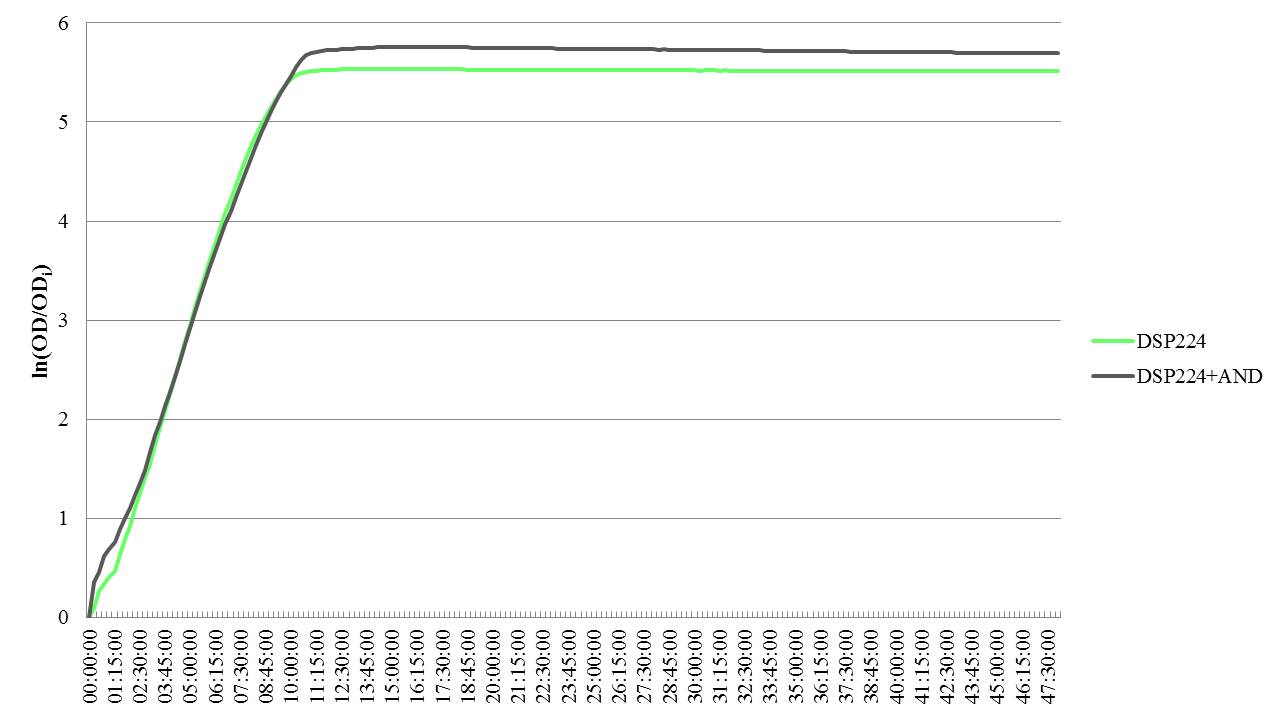
**

**Supplementary Figure 1. Exposure of *C. glabrata* cells to anidulafungin.** Cells from the DSP2 (wild-type) and DSP224 (*FKS1*-S629P) isolates were treated with 0.06 µg/mL of anidulafungin (AND). 4 x 10^4^ cells were deposited in triplicate in the wells of 96-well plates and ODs were measured every 15 min for 48 h. Growth curves were derived from the raw OD curves and are shown as logarithmic curves, *i.e.* Ln (OD/ODi), where ODi is the initial OD.

**
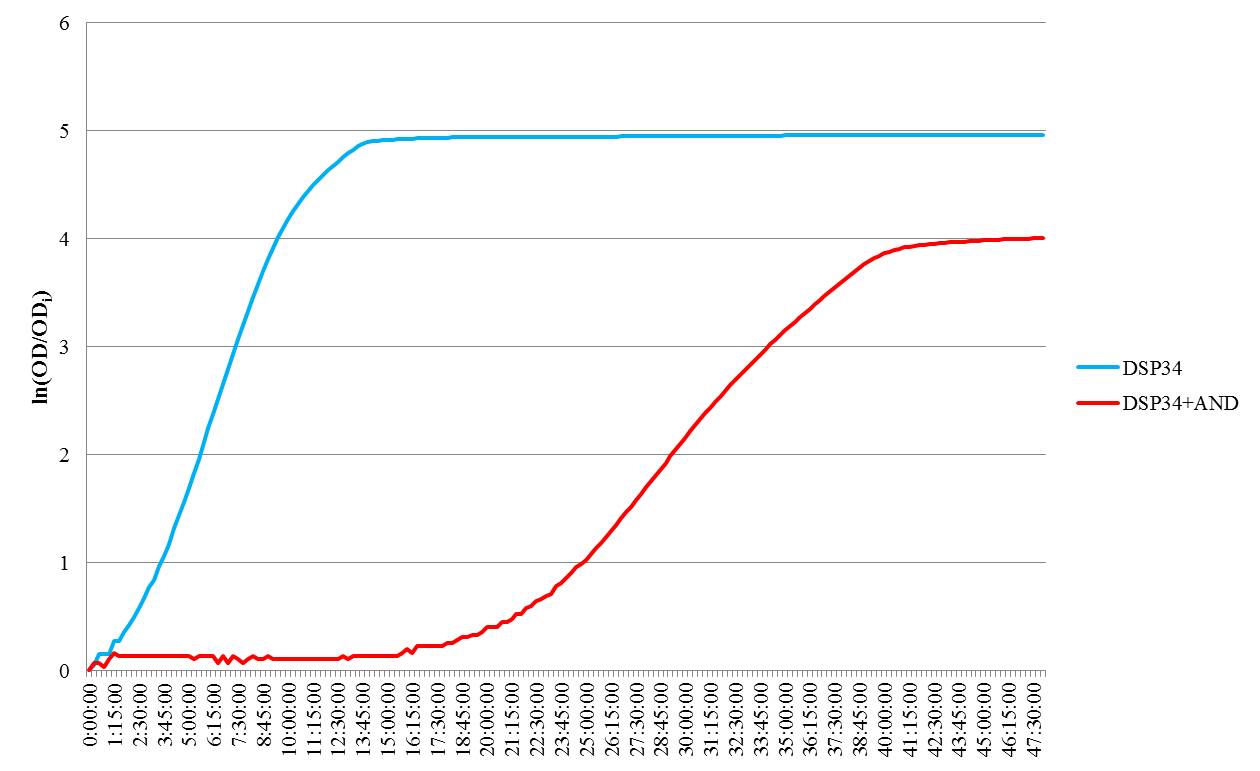
**

**
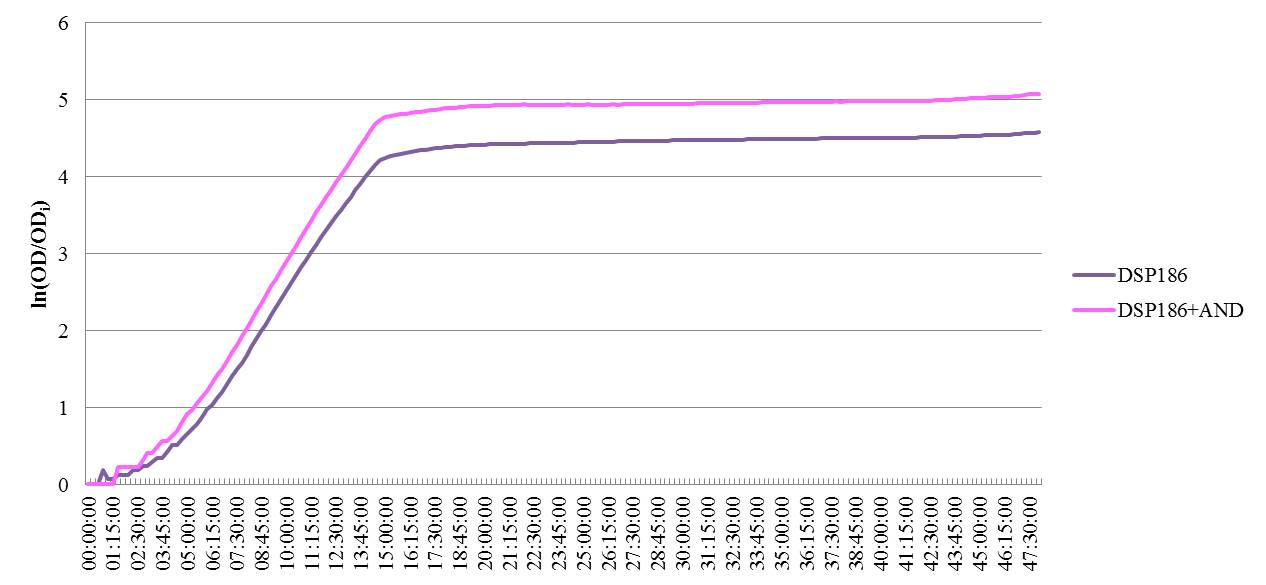
**

**Supplementary Figure 2. Exposure of *C. glabrata* cells to anidulafungin.** Cells from the DSP34 (*FKS2*-P667T) and DSP186 (*FKS2*-S663P) isolates were treated with 0.06 µg/mL of anidulafungin (AND). 4 x 10^4^ cells were deposited in triplicate in the wells of 96-well plates and ODs were measured every 15 min for 48 h. Growth curves were derived from the raw OD curves and are shown as logarithmic curves, *i.e.* Ln (OD/ODi), where ODi is the initial OD.

| **Isolates** | | Molecular |  | Anidulafungin | | |  | Molecular | | | |  | Fluconazole | | |
| --- | --- | --- | --- | --- | --- | --- | --- | --- | --- | --- | --- | --- | --- | --- | --- |
|  |  | **characterization** |  |  | | |  | characterization | | | |  |  | | |
|  |  |  |  | CLSI | | **MS-AFST** |  | **Gene expression (fold increase)** | | | |  | **CLSI** | | **MS-AFST** |
|  |  | ***FKS1*/*FKS2* phenotype** |  | **MIC (µg/mL)** | Category |  |  | **CgCDR1** | ***CgCDR2*** | ***CgSNQ2*** | ***CgERG11*** |  | **MIC (µg/mL)** | Category |  |
| DSP | 1 | WT |  | 0.06 | S | S |  |  |  |  |  |  | 16 | S-DD | S |
| DSP | 2 | WT |  | 0.06 | S | S |  |  |  |  |  |  | 16 | S-DD | S |
| DSP | 3 | WT |  | 0.03 | S | S |  |  |  |  |  |  | 16 | S-DD | S |
| DSP | 4 | WT |  | 0.06 | S | S |  |  |  |  |  |  | 32 | S-DD | S |
| DSP | 5 | WT |  | 0.06 | S | S |  |  |  |  |  |  | 8 | S | S |
| DSP | 6 | WT |  | 0.06 | S | S |  |  |  |  |  |  | 32 | S-DD | S |
| DSP | 7 | WT |  | 0.06 | S | S |  |  |  |  |  |  | 8 | S | S |
| DSP | 8 | WT |  | 0.06 | S | S |  |  |  |  |  |  | 32 | S-DD | S |
| DSP | 9 | WT |  | 0.06 | S | S |  |  |  |  |  |  | 8 | S | S |
| DSP | 11 | WT |  | 0.06 | S | S |  |  |  |  |  |  | 16 | S-DD | S |
| DSP | 15 | WT |  | 0.06 | S | S |  | 56.97 | 2.30 |  | 2.84 |  | >64 | R | R |
| DSP | 17 | WT |  | 0.06 | S | S |  | 111.50 | 5.02 |  | 5.17 |  | >64 | R | R |
| DSP | 18 | WT |  | 0.06 | S | S |  | 41.67 |  | 2.25 |  |  | >64 | R | R |
| DSP | 22 | WT |  | 0.06 | S | S |  | 104.07 | 3.07 |  | 3.39 |  | >64 | R | R |
| DSP | 30 | Fks2p-S663P |  | 2 | R | R |  |  |  |  |  |  | 8 | S | S |
| DSP | 31 | WT |  | 0.06 | S | S |  | 89.99 | 2.92 |  | 4.03 |  | >64 | R | R |
| DSP | 32 | Fks2p-D666G |  | 2 | R | R |  |  |  |  |  |  | 16 | S-DD | S |
| DSP | 33 | Fks2p-D666E |  | 0.25 | I | S |  |  |  |  |  |  | 32 | S-DD | S |
| DSP | 34 | Fks2p-P667T |  | 1 | R | S |  | 96.01 | 4.49 |  |  |  | >64 | R | R |
| DSP | 38 | Fks1p-F625S |  | 1 | R | R |  |  |  |  |  |  | 32 | S-DD | S |
| DSP | 39 | Fks1p-S629P |  | 2 | R | R |  | 99.09 | 4.09 |  | 2.47 |  | >64 | R | R |
| DSP | 40 | WT |  | 0.06 | S | S |  | 83.61 | 2.25 |  | 3.57 |  | >64 | R | R |
| DSP | 42 | WT |  | 0.06 | S | S |  | 119.13 | 2.91 |  | 5.29 |  | >64 | R | R |
| DSP | 56 | WT |  | 0.12 | S | S |  | 75.02 |  |  |  |  | >64 | R | R |
| DSP | 64 | WT |  | 0.12 | S | S |  | 119.22 | 4.13 | 2.27 | 20.60 |  | >64 | R | R |
| DSP | 155 | Fks2p-F659V |  | 1 | R | S |  |  |  |  |  |  | 4 | S | S |
| DSP | 170 | Fks1p-F625S |  | 2 | R | R |  | 34.80 |  |  | 8.59 |  | 64 | R | R |
| DSP | 180 | Fks2p-S663P |  | 0.25 | I | S |  |  |  |  |  |  | 16 | S-DD | S |
| DSP | 182 | WT |  | 1 | R | R |  |  |  |  |  |  | 8 | S | S |
| DSP | 186 | Fks2p-S663P |  | 4 | R | R |  | 54.69 |  |  |  |  | >64 | R | R |
| DSP | 220 | Fks2p-S663P |  | 1 | R | S |  |  |  |  |  |  | 8 | S | R |
| DSP | 221 | Fks2p-S663P |  | 1 | R | S |  | 126.77 | 4.76 | 2.38 |  |  | >64 | R | S |
| DSP | 224 | Fks1p-S629P |  | 4 | R | R |  | 32.85 |  |  |  |  | >64 | R | R |
| DSP | 235 | Fks2p-R665S |  | 0.12 | S | S |  | 55.98 |  |  |  |  | >64 | R | R |
| DSP | 238 | WT |  | 0.12 | S | S |  |  |  |  |  |  | 8 | S | S |
| DSP | 249 | Fks2p-S663P |  | 4 | R | R |  | 124.60 |  |  |  |  | >64 | R | R |
| DSP | 254 | WT |  | 0.12 | S | S |  |  |  |  |  |  | 16 | S-DD | S |
| DSP | 255 | Fks1p-D632E |  | 1 | R | R |  | 4.71 | 3.06 |  | 3.52 |  | 64 | R | R |
| DSP | 259 | Fks2p-F659V |  | 1 | R | S |  | 6.16 | 4.05 |  |  |  | 64 | R | S |
| DSP | 260 | Fks1p-D632E |  | 1 | R | R |  | 230.35 |  |  |  |  | >64 | R | R |
| DSP | 265 | Fks2p-S663P |  | 2 | R | S |  |  |  |  |  |  | 16 | S-DD | S |
| DSP | 282 | Fks2p-R665G |  | 0.25 | I | S |  | 17.47 | 2.17 |  |  |  | >64 | R | R |
| DSP | 307 | Fks2p-S663P |  | 1 | R | S |  |  |  |  |  |  | 8 | S | S |
| DSP | 314 | Fks2p-P667T |  | 0.25 | I | S |  |  |  |  |  |  | 16 | S-DD | S |
| DSY | 562 | WT |  | 0.03 | S | S |  |  |  |  |  |  | 8 | S | S |
| SFY | 98 | WT |  | ≤0.016 | S | S |  |  |  |  |  |  | 2 | S | S |
| SFY | 99 | WT |  | ≤0.016 | S | S |  | 24 | 3.5 |  |  |  | >64 | R | R |
| SFY | 100 | WT |  | ≤0.016 | S | S |  |  |  |  |  |  | 8 | S | S |
| SFY | 101 | WT |  | ≤0.016 | S | S |  | 32 |  |  |  |  | >64 | R | R |
| SFY | 102 | WT |  | ≤0.016 | S | S |  |  |  |  |  |  | 8 | S | S |
| SFY | 103 | WT |  | ≤0.016 | S | S |  | 15.5 | 360 |  |  |  | >64 | R | R |
| SFY | 104 | WT |  | ≤0.016 | S | S |  |  |  |  |  |  | 8 | S | S |
| SFY | 105 | WT |  | ≤0.016 | S | S |  | 4.5 | 32 |  |  |  | >64 | R | R |
| SFY | 106 | WT |  | ≤0.016 | S | S |  |  |  |  |  |  | 4 | S | S |
| SFY | 107 | WT |  | ≤0.016 | S | S |  | 4 |  |  |  |  | >64 | R | R |
| SFY | 108 | WT |  | ≤0.016 | S | S |  |  |  |  |  |  | 8 | S | S |
| SFY | 109 | WT |  | ≤0.016 | S | S |  | 5.5 |  |  |  |  | >64 | R | R |
| SFY | 110 | WT |  | ≤0.016 | S | S |  |  |  |  |  |  | 8 | S | S |
| SFY | 111 | WT |  | ≤0.016 | S | S |  | 12 |  |  |  |  | >64 | R | R |
| SFY | 112 | WT |  | ≤0.016 | S | S |  |  |  |  |  |  | 8 | S | S |
| SFY | 113 | WT |  | 0.03 | S | S |  | 192 | 4 |  |  |  | >64 | R | R |
| UCSC | 1 | WT |  | 0.03 | S | S |  |  |  |  |  |  | 4 | S | S |
| UCSC | 2 | WT |  | 0.03 | S | S |  |  |  |  |  |  | 8 | S | S |
| UCSC | 3 | WT |  | 0.03 | S | S |  |  |  |  |  |  | 4 | S | S |
| UCSC | 4 | WT |  | 0.03 | S | S |  |  |  |  |  |  | 4 | S | S |
| UCSC | 5 | WT |  | 0.03 | S | S |  |  |  |  |  |  | 4 | S | S |
| UCSC | 6 | WT |  | 0.03 | S | S |  |  |  |  |  |  | 4 | S | S |
| UCSC | 7 | WT |  | 0.03 | S | S |  |  |  |  |  |  | 4 | S | S |
| UCSC | 8 | WT |  | 0.03 | S | S |  |  |  |  |  |  | 8 | S | S |
| UCSC | 9 | WT |  | 0.03 | S | S |  |  |  |  |  |  | 4 | S | S |
| UCSC | 10 | WT |  | 0.06 | S | S |  | 9.97 |  |  |  |  | 64 | R | R |
| UCSC | 11 | WT |  | 0.03 | S | S |  | 33.75 |  |  |  |  | 64 | R | R |
| UCSC | 12 | WT |  | 0.03 | S | S |  | 33.38 |  |  |  |  | 64 | R | R |
| UCSC | 13 | WT |  | 0.03 | S | S |  | 163.66 | 2.98 | 2.22 |  |  | 64 | R | R |
| UCSC | 14 | WT |  | 0.03 | S | S |  | 265.98 | 4.93 | 2.34 |  |  | >64 | R | R |
| UCSC | 15 | WT |  | 0.03 | S | S |  | 32.37 |  |  |  |  | >64 | R | R |
| UCSC | 16 | WT |  | 0.06 | S | S |  | 64.03 |  |  |  |  | >64 | R | R |
| UCSC | 17 | WT |  | 0.06 | S | S |  | 179.39 |  | 4.43 | 8.39 |  | >64 | R | R |
| UCSC | 18 | WT |  | 0.03 | S | S |  | 49.44 | 2.21 |  | 3.21 |  | >64 | R | R |
| UCSC | 19 | WT |  | 0.12 | S | S |  | 37.63 |  |  | 2.9 |  | >64 | R | R |

**Supplementary Table 1. Phenotypic/semi-molecular antifungal susceptibility testing and resistance genotype/mechanism characterization for the 80 *C. glabrata* isolates included in the study.** The CLSI method and the MS-AFST assay were performed with anidulafungin and fluconazole antifungal agents, as detailed in the text, using isolates well characterized with respect to the antifungal resistance and designated as follows: DSP, culture strain collection of the New Jersey Medical School, Newark, NJ, USA; DSY and SFY, culture strain collections of the University Hospital Centre, Lausanne, Switzerland; UCSC, culture strain collection of the Catholic University of the Sacred Heart, Rome, Italy. Ten of 80 *C. glabrata* isolates, representative of wild-type (DSY562, UCSC1, UCSC10, UCSC11, UCSC12) and non-wild-type (DSP32, DSP38, DDSP39, UCSC14, UCSC16) phenotypes against fluconazole or anidulafungin were used in the first stage of the study.

|  | **Method** | **No. of isolates (NWT/WT) tested** | **No. (%) of isolates for which test results were in the indicated category** | | |
| --- | --- | --- | --- | --- | --- |
| **Antifungal drug** |  |  | **CA** | **VMEs** | **MEs** |
| Anidulafungin | MS-AFST | 80 (22/58) | 68/80 (85.0) | 11/80 (13.8) | 1/80 (1.3) |
|  | CLSI | 80 (22/58) | 78/80 (97.5) | 1/80 (1.3) | 1/80 (1.3) |
|  |  |  |  |  |  |
| Fluconazole | MS-AFST | 80 (39/41) | 77/80 (96.2) | 2/80 (2.5) | 1/80 (1.3) |
|  | CLSI | 80 (39/41) | 80/80 (100) | 0/80 | 0/80 |

**Supplementary Table 2. Performances of MS-AFST and the CLSI method compared to resistance molecular analysis for the 80 *C. glabrata* isolates tested against two antifungal drugs.** Results from both MS-AFST and the CLSI method were analysed in comparison with the results from the genotype/resistance mechanism analysis. Percentages of categorical agreement (CA) between the test results were calculated. Very major errors (VMEs) were identified when non-wild-type (NWT) isolates (*i.e.* for anidulafungin, isolates harbouring *FKS1*/*FKS2* HS1 mutations or for fluconazole, isolates overexpressing *CDR* genes) were detected as absent by MS-AFST or the CLSI method. Major errors (MEs) were identified when wild-type (WT) isolates were detected as present by MS-AFST or the CLSI method.

| **Isolate designation** | **MIC (µg/mL)** | **CLSI category** | *MS-AFST assay result at* | | | | **MPCC determined at** | **MIC (µg/mL)** | **CLSI category** | **MS-AFST assay result a**  **T** | | | **MPCC determined at** |
| --- | --- | --- | --- | --- | --- | --- | --- | --- | --- | --- | --- | --- | --- |
|  |  |  | ***3 h*** | ***6 h*** | ***9 h*** | ***12 h*** | **15 h** |  |  | **3 h** | **6 h** | **9 h** | **15 h** |
|  | ***Anidulafungin*** | | | | | | | **Fluconazole** | | | | | |
| DSP33 | 0.25 | I | ***S*** | ***S*** | ***R*** | ***-*** | 0.25 |  |  |  |  |  |  |
| DSP34 | 1 | R | ***S*** | ***R*** | ***-*** | ***-*** | 0.5 |  |  |  |  |  |  |
| DSP155 | 1 | R | ***S*** | ***S*** | ***R*** | ***-*** | 2 |  |  |  |  |  |  |
| DSP180 | 0.25 | I | ***S*** | ***S*** | ***S*** | ***R*** | 0.25 |  |  |  |  |  |  |
| DSP220 | 1 | R | ***S*** | ***R*** | ***-*** | *-* | 2 | 8 | S | R | **S** | - | 8 |
| DSP221 | 1 | R | ***S*** | ***S*** | ***R*** | *-* | 2 | >64 | R | S | S | **R** | 64 |
| DSP259 | 1 | R | ***S*** | ***S*** | ***S*** | ***R*** | 0.5 | 64 | R | S | **R** | - | 64 |
| DSP265 | 2 | R | ***S*** | ***S*** | ***S*** | ***R*** | 8 |  |  |  |  |  |  |
| DSP282 | 0.25 | I | *S* | *S* | *S* | *S* | 0.25 |  |  |  |  |  |  |
| DSP307 | 1 | R | *S* | *S* | *S* | ***R*** | 1 |  |  |  |  |  |  |
| DSP314 | 0.25 | I | *S* | *S* | *S* | *S* | 0.25 |  |  |  |  |  |  |

**Supplementary Table 3. Resolution of discrepancies for *C. glabrata* isolates which were misclassified by the MS-AFST assay performed with a 3-h antifungal drug exposure.** To attempt to give an explanation for discrepancies with the CLSI susceptible (S)/nonsusceptible (intermediate, I; resistant, R) category, MS-AFST assays were performed with the 11 isolates at the indicated times of incubation with anidulafungin or fluconazole. As shown, all the isolates except for DSP282 and DSP314 were classified as R to anidulafungin at a 6-h (2 isolates), 9-h (3 isolates) or 12-h (4 isolates) drug exposure, whereas all (3 of the 11) isolates were classified as S (1 isolate) or R (2 isolates) to fluconazole at a 6-h (2 isolates) or 9-h (1 isolate) drug exposure. The correct MS-AFST classification results achieved following prolonged drug-exposure times are marked in bold. In the meanwhile, MPCC values were determined, using the previously developed version of MS-AFST assay, for all the isolates and showed excellent (100%) categorical agreement with the CLSI MIC values.
